# *Helicobacter pylori* Diagnostic Testing Accuracy in a High-Prevalence Native American Population of Northern Arizona

**DOI:** 10.3390/microorganisms13081920

**Published:** 2025-08-18

**Authors:** Heidi E. Brown, Laura Pauli, Rishi Dholakia, Joseph Gunderson, Julia Jernberg, Priscilla R. Sanderson, Robin B. Harris, Fernando P. Monroy

**Affiliations:** 1 Department of Epidemiology and Biostatistics, Mel and Enid Zuckerman College of Public Health, 1295 N Martin Ave, Tucson, AZ 85724, USA; rbharris@arizona.edu; 2 Winslow Indian Health Care Center, 500 North Indiana Avenue, Winslow, AZ 86047, USA; laura.pauli@wihcc.org (L.P.); rishi.dholakia@gmail.com (R.D.); 3 Department of Medicine, College of Medicine, The University of Arizona, 1501 N Campbell Ave, Tucson, AZ 85724, USA; jrgunderson@arizona.edu (J.G.); jbj1@arizona.edu (J.J.); 4 Department of Health Sciences, College of Health and Human Services, Northern Arizona University, SAS (Bldg 60), 1100 South Beaver Street, P.O. Box 15095, Flagstaff, AZ 86011, USA; priscilla.sanderson@nau.edu; 5 Department of Biological Sciences, College of the Environment, Forestry and Natural Sciences, Northern Arizona University, 617 South Beaver Street, Flagstaff, AZ 86011, USA; 6 Pathogen and Microbiome Institute, Northern Arizona University, Flagstaff, AZ 86011, USA

**Keywords:** diagnostics, campylobacter-like organism test (CLOtest), histopathology, *H. pylori*, gastric cancer

## Abstract

*Helicobacter pylori* (*H. pylori)* is a common gastric pathogen and a leading cause of non-cardia gastric cancers. Known determinants can affect the diagnostic accuracy of invasive clinical methods for *H. pylori* diagnosis. The objective of this study was to determine the diagnostic accuracy of the CLOtest, a rapid urease test, and the histopathologic examination compared with polymerase chain reaction (PCR) in esophagogastroduodenoscopy patients from a population with high prevalence and other risk factors that may influence diagnostic accuracy. From 2018 to 2022, patients were recruited from a medical care center serving the southwestern Navajo Nation. Summary statistics were calculated using PCR as the comparator to the CLOtest and histopathologic examination. Among the 466 study participants, 27.1% (95% CI 22.9, 31.7%) tested positive for *H. pylori* using PCR to detect pathogen DNA. Sensitivity was lowest for the CLOtest (57.0%; 95% CI 45.8, 67.6) and highest for the combination the CLOtest and histopathology (72.2%; 95% CI 62.8, 80.4). Patient history of infection or possible GI bleeding influenced sensitivity by over 5%. In high *H. pylori* prevalence areas, emphasis should be placed on ensuring adequate treatment of suspected positive infections as false-positive results were rare. Including a more sensitive test might reduce the number of individuals falsely classified as *H. pylori* negative.

## 1. Introduction

*Helicobacter pylori* (*H. pylori*) is a common gastric pathogen and a leading cause of non-cardia gastric cancers [1]. The global prevalence of *H. pylori* ranges from 19% in developed countries to 88% in developing nations [2]. The United States estimated that *H. pylori* prevalence is 35%. However, a much higher prevalence is observed among Native Americans [2]. Among members of the Navajo Nation, active *H. pylori* prevalence has been estimated to be as high as 56–70%, which likely contributes to gastric cancer rates that are 3–4 times higher compared to non-Hispanic Whites in Arizona [3,4,5]. Gastric cancer is an outcome in approximately 9% of persons with *H. pylori* infection globally [6].

The detection of *H. pylori* infection is established via invasive and non-invasive techniques with varying sensitivities, specificities, and predictive values that fluctuate with disease prevalence, patient risk factors, or concurrent medication use. Common non-invasive diagnostic methods include the urea breath test (UBT), serology, and stool antigen assays. The UBT is among the most commonly used non-invasive diagnostic procedures, which identifies active infection by detecting a labeled carbon isotope in the patient’s expired air, produced as a byproduct of urea hydrolysis by *H. pylori* urease. Invasive diagnostic tests involve procuring gastric mucosal samples by swabbing, brushing, or during an esophagogastroduodenoscopy (EGD). Common clinical methods include the Rapid urease tests (RUTs, e.g., the Campylobacter-like organism test (CLOtest)) and histopathological evaluation. Additionally, polymerase chain reaction (PCR) testing can be used to detect *H. pylori* DNA, but it may require collaboration with research partners, limiting its use in clinical settings.

*H. pylori* detection tests have known determinants that impact their diagnostic accuracy. Rapid urease tests (e.g., a CLOtest) have been noted to return frequent false negative results with recent use of antibiotics, bismuth-containing compounds, proton pump inhibitors (PPIs), and the presence of intestinal metaplasia [7], atrophic gastritis [8], or upper gastrointestinal (GI) bleeding [9]. The sensitivity of histopathological analysis is similarly reduced by upper GI bleeding [10,11], atrophic gastritis [12], recent PPI therapy, or the presence of intestinal metaplasia [13]. Older studies compared RUTs with histopathology and showed somewhat improved sensitivity in the setting of upper GI bleeding [10,14,15]. Additionally, the accuracy of histopathologic evaluation depends on the skills of the microscopist or pathologist [13].

The Navajo Nation population exhibits unique environmental risk factors that may influence the accuracy of existing *H. pylori* diagnostic tests. Harris et al. [3] found a significant association between a lack of access to regulated water and a higher prevalence of *H. pylori*. Over 30% of Navajo members lack access to public water systems, and unregulated water sources may exceed the maximum contaminant levels of arsenic and uranium outlined in the Safe Drinking Act [16]. Chronic arsenic exposure has been tied to an increased risk for GI bleeding, inflammation, and malignancy [17,18]. Furthermore, to ensure stability of the enzyme and other components, CLOtest kits require storage at temperatures between 2 °C and 8 °C, which may not be reliably achievable under Northern Arizona climate conditions during the summer months [19].

Given the high prevalence of *H. pylori* among members of the Navajo Nation, it is imperative to ensure accurate and reliable diagnosis. Determining the true presence or absence of this carcinogenic organism is crucial for optimal treatment and improved health outcomes. This study aimed to evaluate the diagnostic accuracy of the CLOtest and histopathology in comparison to PCR testing among Navajo Nation members who were determined to require an esophagogastroduodenoscopy (EGD) test for medical reasons.

## 2. Materials and Methods

### 2.1. Setting and Subjects

For this cross-sectional study, we recruited 466 patients undergoing EGD at Winslow Indian Health Care Center (WIHCC) between 29 January 2018, and 10 August 2022. WIHCC provides medical care to eight chapters in the southwestern Navajo Nation. All adult patients scheduled for an EGD during the study period who reported Navajo as their tribal affiliation were eligible to participate. A packet of information about the study was provided during check-in to the clinic for the procedure, including copy of the consent form. The Navajo clinic nurse or the physician performing the procedure was present to answer questions. Individuals gave written informed consent. All participants provided informed consent for the abstraction of their medical records and the acquisition of two additional gastric biopsy samples during their elective EGD. After consent, samples were collected by the physician and medical record abstractions performed by the clinic nurse. Participants were only identified through a participant ID. Links between participant name and research data were not provided to the research lab for sample testing or data analysis.

### 2.2. Sample Testing

All biopsies underwent PCR testing for the detection of active *H. pylori* infection and the identification of virulence genes. Indeterminate results were excluded from the analysis. Three diagnostic tests were used to assess the status of *H. pylori* infection. WIHCC performed the CLO Rapid Urease Test (Avanos, GA, USA). One pair of gastric biopsy samples was sent to a laboratory contracted by WIHCC to perform histopathological examination and tested using immunohistochemical (IHC) staining; the precise techniques and methods may have varied depending on the pathologist’s clinical judgment. PCR testing was performed by Northern Arizona University (NAU) research partners between 29 January 2018, and 21 April 2023, and described elsewhere [20]. The two additional research biopsy samples were separately placed in 400 µL RNAlater (Sigma Chemical Co., St. Louis, MO, USA) filled tubes and then transported to the NAU laboratory. Pathogen DNA was isolated using the FastDNA Spin Kit (MP Biomedicals, LLC, Solon, OH, USA) and the FastPrep 24 instruments (MP Biomedicals LLC) as described by the manufacturer. Positive and negative controls were included in each reaction. In some cases, the ~519 bp-amplified product was separated by electrophoresis in 1% agarose gels, followed by SYBR Green staining and analysis under an ultraviolet (UV) light. The 16S rDNA gene was used with *H. pylori* 26695 (ATCC 700392) and *H. pylori* 60190 (ATCC 49503) strains as reference isolates.

### 2.3. Medical Record Abstraction

Medical record data—including demographic information, endoscopy indication, and previous *H. pylori* testing information—were abstracted by WIHCC staff members between 15 September 2018 and 22 August 2022. The medical record data and biopsy samples were de-identified and linked only by study participant ID, with the research team not having access to the linkage. Body Mass Index (BMI) was calculated from recorded height and weight values and classified by WHO categories [21].

### 2.4. Diagnostic Risk Factors

*H. pylori* population-specific prevalence, recent treatment for *H. pylori* infection, and the presence of blood in the gastric environment are established factors influencing the outcome of the diagnostic tests described above. For the estimation of predictive values, prevalence was estimated using published values for this population undergoing EGD [20], and the larger community from which these patients arise [3]. Samples were classified as being from patients with a suspected history of *H. pylori* infection when the record abstraction included treatment for the infection or when the indication for the EGD was listed as eradication check. Samples were classified as being from patients with suspected blood in the gastric environment-based signs or symptoms of GI bleeding in the record abstractions—blood in vomit or stool, a known upper GI bleed, or anemia significant enough to indicate endoscopy.

### 2.5. Data Analysis

We used real-time PCR as the gold standard, as it is highly sensitive and specific, and is recommended as a gold standard where laboratory capacity exists [22]. However, it is considered experimental and is not widely available for clinical use [23,24]. There is no consensus regarding a universal gold standard for comparing direct (e.g., histopathology, CLOtest, or culture) and indirect (antibody in stool or urine, UBT, or stool antigen tests) diagnostic testing [25]. As a clinical test-and-treat diagnostic tool, the noninvasive UBT is considered the gold standard and is appropriate for noninvasive confirmation of eradication or reinfection in high-prevalence populations [26,27,28]. Among invasive clinical diagnostic tools, histopathological examination is considered the gold standard for clinical diagnosis using biopsy samples [28].

Sensitivity, specificity, positive predictive value (PPV), and negative predictive value (NPV), Receiver Operating Curves (ROC), percent agreement, and kappa coefficients were calculated separately for the CLOtest and histopathology against PCR as the gold standard [22]. We also evaluated the instances when a sample was positive, as well as when either the CLOtest or histopathology test was positive. We assumed 23% *H. pylori* prevalence in this population of patients undergoing EGD [20]. Analyses were stratified by risk factors that affect these diagnostic tests, specifically medical record notation of prior *H. pylori* infection history and presence of blood in the gastric environment. We did not further stratify by known risk factors such as age or sex.

Analyses were performed using Stata v17.0 (College Station, TX, USA). Demographic characteristics were compared by *t*-test and chi-square with Fisher’s exact to correct for small counts. The diagt command was used to summarize the diagnostic test performance. Cohen’s Kappa assessed interrater reliability between the tests according to guidelines suggested by Landis and Koch [29], where kappa < 0.00 indicated poor agreement, 0.00–0.20 was slight, 0.21–0.40 was fair, 0.41–0.60 was moderate, 0.61–0.80 was substantial, and 0.81–1.00 indicated almost perfect agreement. Only 117 samples would have been necessary to detect a medium effect at alpha = 0.05 and a power = 0.9.

### 2.6. Ethical Approvals

Prior to beginning sample collection, formal support tribal resolutions were obtained for this research from participating chapters, associated Navajo Agency Council areas, and Winslow Indian Health Care Center. The protocol and consent forms were approved by the Navajo Nation Human Research Review Board (NNHRRB; NNR-16-26). The study also was reviewed and approved by the NAU IRB (NAU: 819500-1). All data were deidentified with links broken prior to sending samples to NAU the study was deemed exempt. An earlier draft of this manuscript was approved by the NNHRRB 18 June 2024, with notification received 17 January 2025.

### 2.7. Patient and Public Involvement

The inception of this project was the observation of a clinical partner of the high rates of gastric cancer in his clinic and subsequent discussions about the diagnostic tests used to detect *H. pylori*, a known risk factor for developing gastric cancer. As part of all work on the Navajo Nation, we presented the project for approval and comments to community members at chapter meetings in the area before beginning the project. We also report the findings to the communities and continue to do so.

## 3. Results

**Figure 1:**
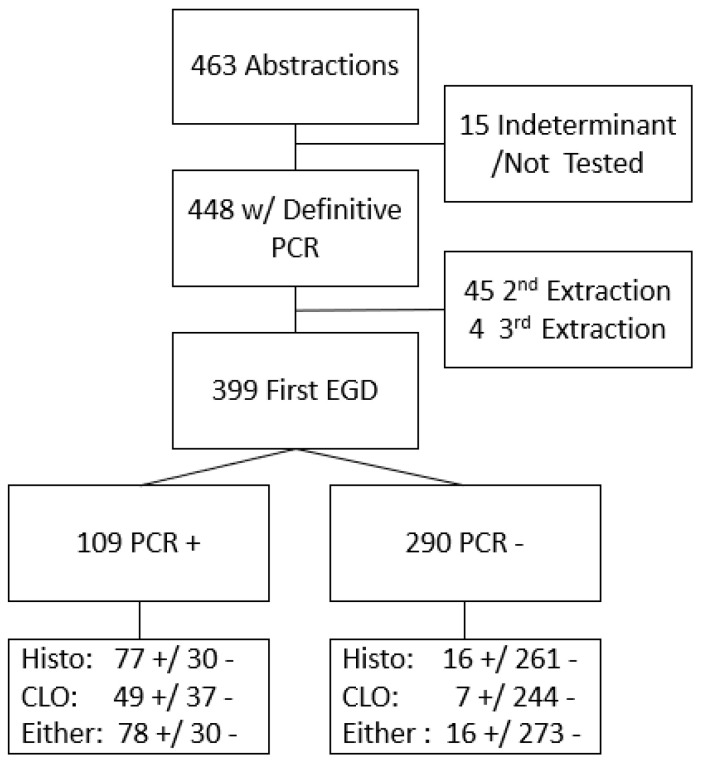
Flow of participants through PCR and compared by Histopathology (Histo) or Campylobacter-like organism test (CLOtest) diagnostic testing result either alone or together. + indicates a positive result; - indicates a negative result.

Among 463 participating patients with complete records and biopsy samples, PCR results were definitive for 4448 (15 had indeterminate results and were excluded). Of these 448 samples, 399 were identified as originating from their earliest endoscopy during the study period (Figure 1). Of these 399 patients, the CLOtest was performed in 337 cases, histopathologic examination in 384, and a combination of either the CLOtest or histopathology in 397 patients. *H. pylori* infection prevalence was 27.1% (95% CI 22.9, 31.7%) based on PCR results. Using histopathology results only, prevalence was similar (24.2%; 95% CI 20.2%, 28.8%). However, the prevalence estimate based on the CLOtest was lower (16.6%, 95% CI 13.0%, 21.0%). The US prevalence is estimated at 30%, and our prior work in this same population estimated the community prevalence of active infection to be 56% using UBT [3,30].

Table 1 presents a comparison of participants by demographic and clinical indicators. Patients testing negative for *H. pylori* were older (57.6 years) than those testing positive (52.5 years). This effect was more pronounced among patients with a suspected history of *H. pylori* infection (59.0 years versus 52.6 years for those testing negative and positive, respectively, *p* < 0.01). Males had a higher percentage of positive test results (*p* < 0.01).

**Figure 2:**
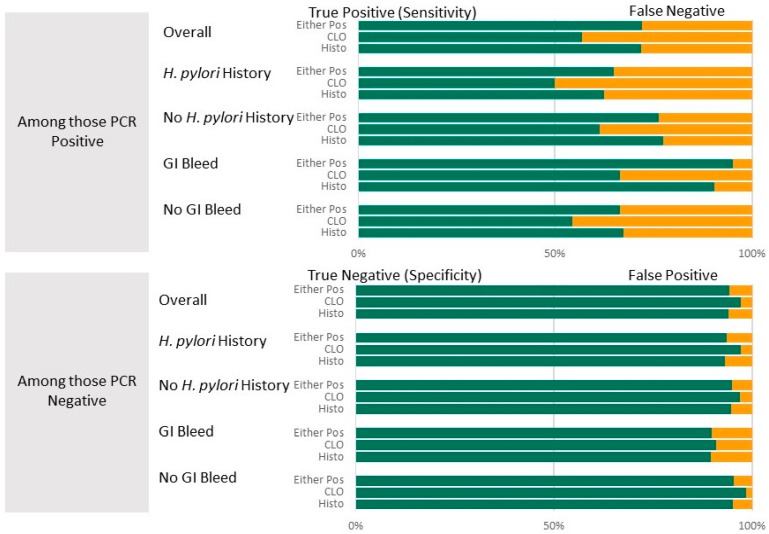
Diagnostic accuracy (top panel: Sensitivity shown in green (with false negatives shown in yellow; lower Panel: Specificity shown in green with false positives shown in yellow)) of the CLOtest and histopathology diagnostic tests overall and by suspected history of *H. pylori* or GI bleed as EGD indicators. PCR test results were the gold standard.

Table 2 presents summary statistics for diagnostic test results (CLOtest, histopathology, and combination). The table summarizes diagnostic accuracy for all participants. It is then stratified by whether or not a history of *H. pylori* was suspected and whether or not a suspicion of GI bleed was an indication for the EGD. Both the CLOtest and histopathology, individually and in combination, have high specificity (94.2–97.2%) and high NPV (88.3–91.9%) compared to the PCR results. Sensitivity was lowest for the CLOtest (57.0%) and highest for the combination (72.2%), while PPV was lowest for histopathology (78.8%) and highest for the CLOtest (85.9%). These results are visualized in Figure 2. There was substantial agreement between the clinical and PCR results, with Kappa values between 0.61 and 0.70.

**Stratifying by suspected history of *H. pylori*.** Among those with a suspected history of *H. pylori* infection in the medical record, the ability to detect non-infected individuals remained high (specificity 93.3–97.3%), while the ability to detect individuals with an infection was reduced (sensitivity decreased by 7–9.5%) compared to the overall rate. Predictive values also remained near 80%, although slightly reduced from the overall predictive values for patients with a history of infection: PPV for a positive histopathology decreased by 5.1%, and by 4.4% for the combination. In contrast, compared with the overall, when restricting the comparison to those without a history of *H. pylori,* the test statistics improved (sensitivity by 4.3–5.6%) or remained nearly the same (average change for specificity, NPV, and PPV was 1.1%; Table 2).

**Stratifying by Suspected GI bleed as EGD indication.** Among those with suspicion of GI bleed as an indication for the EGD, the ability to detect an infected individual increased compared with the overall ability for each test individually. The greatest increase was improved sensitivity by 23% when using the combination, but histopathology alone yielded an 18% improvement. Detecting a non-infected individual decreased by about 5% across all tests. However, few (<70) patients were identified as having a possible GI bleed, and the resulting confidence intervals are wide. In contrast, compared with the overall, when restricting comparisons to those without GI bleed, sensitivity decreased by about 4%. Specificities, PPV, and NPVs remained within 1–2% of the overall, except for PPV, which increased by 5.9%.

**Demographic and clinical characteristics of false positives and false negatives.** A comparison of demographics between those correctly detected and those missed as cases (sensitivity, Table S1) revealed that the mean age of the 29 participants incorrectly identified as negative was higher, at 57.6 years, compared to the 50.7 years of the 76 participants correctly identified as positive by histopathology (*p* = 0.04). Likewise, the average age of the 36 patients with false-negative results was higher than the mean for the 49 participants correctly identified as positive by the CLOtest (59.6 vs. 49.6, *p* < 0.01). Although the trends were similar, there were no significant demographic differences between true negatives and false positives by either histopathology or the CLOtest (Table S2).

## 4. Discussion

This cross-sectional study aimed to assess two routine invasive clinical diagnostic tests, the CLOtest and histopathology, in comparison to PCR for identifying *H*. *pylori* infection among patients undergoing scheduled EGD within an American Indian-serving clinical setting. Among those patients for whom this was their first EGD within the study period, 27.1% were positive for *H. pylori* by PCR. This is lower than the prevalence of the source population. However, this was expected as the individuals included in the study may have been undergoing treatment for GI conditions (41.4% of participants indicated prior *H. pylori* infection). As others have shown [31], *H. pylori* was less common among female patients (23% positive compared with 37% of males). We found no differences by BMI or smoking status.

The diagnostic tests had a higher capacity to correctly identify negative patients (specificity) and a moderate ability to identify infected patients (sensitivity). The highest agreement (TP + TN)/total: 88.4%) was achieved when the CLOtest or histopathology was used to indicate infection. This means, in a hypothetical setting where the prevalence is nearly 25%, both tests are used. Still, only one needs to be positive to diagnose *H. pylori* infection; among 1000 patients, 70 infected patients would be incorrectly identified as negative. In contrast, 41 patients without the infection would be incorrectly marked as having the infection. The lowest false positive rate (i.e., chance of treating a patient who is not infected) occurs when using the CLOtest alone. Still, the CLOtest alone also returns the highest false negative (108 missed diagnoses in our hypothetical population). Our results suggest that false-positive results are rare with these tests, meaning emphasis should be placed on ensuring adequate treatment of a suspected positive *H. pylori* infection.

Sensitivity decreased across both diagnostic tests among patients with a suspected history of *H. pylori*, which is consistent with prior studies finding that treatment with PPIs and antibiotics can impede *H. pylori* detection [13]. The American College of Gastroenterology recommends two weeks of withheld therapy before testing for eradication [32]. While the data abstraction process did not clarify the timing of each patient’s prior *H. pylori* infection to ascertain if or when they ceased PPI or antibiotic therapy, the observed decrease in sensitivity is expected.

In contrast to prior studies, sensitivity increased for the CLOtest and histopathology among those with a possible GI bleed. Tu et al. [10] found CLOtests and histopathology were less sensitive than non-invasive tests like the UBT among patients with peptic ulcer bleeding (CLOtest: 45.5%, histopathology: 77.2%, and UBT: 95.4%). Further, they found that blood in the antrum had the strongest effect on the CLOtest sensitivity. Sensitivities were higher among those with a suspected GI bleed as an endoscopy indication for the CLOtest and histopathology (66.7% and 90.5%, respectively) than those without GI bleeding (54.4% and 67.4%, respectively). However, we defined a GI bleed indication as anemia significant enough to warrant EGD or blood in the stool or vomit. Specific underlying features to more clearly define this symptomatology were limited. It must be noted that the number of patients with this indication was small (n < 70).

Guidelines for treatment of *H. pylori* include 7 or 14 days of a combination of antibiotics plus a PPI, with a recommendation to consider probiotics [32]. Further, GI upset is commonly reported as an association with treatment, and 21% of respondents in a national survey did not complete *H. pylori* treatment as a result [33]. Given that *H. pylori* antibiotic resistance rates are rising [34], the high prevalence of *H. pylori* and diagnostic specificity suggest a lower risk of unwarranted treatments. Regional antibiotic susceptibility data for *H. pylori* are scarce [32]; although high presence of antibiotic resistance mutations in amplified *H. pylori* DNA has been identified in this population (38% for clarithromycin and 94% for metronidazole) [35]. Inquiring about previous antibiotic use may help clinicians select treatment regimens more effectively [36]. Our results indicate that *H. pylori* is more frequently underdiagnosed (low sensitivity) than falsely diagnosed (high specificity). These findings suggest that clinicians within the Navajo population should favor treating patients with diagnostic indications. Furthermore, ensuring strong patient support and follow-up to promote compliance with antibiotic therapy remains critical to mitigating the risks of undertreated infections and antibiotic resistance.

Our study has limitations. Although WIHCC is the region’s primary clinic for Navajo citizens, this study included a sample of patients who underwent endoscopy and elected to participate in this study. Thus, it may not be representative of the community with respect to symptomology, treatment, and access to care. Additionally, the record abstraction and medication review were not comprehensive for potential treatments in other locations and whether medications were described in the record. These issues may have led to some misclassification regarding suspected *H. pylori* history or GI bleeding. However, we included only patient data from their first visit to minimize the inclusion of patients who may have been post-*H. pylori* treatment. Another possible limitation may have been the collection of a single antral and fundal specimen for the research arm of this study. For the assessment of *H. pylori*, atrophic gastritis, and gastric intestinal metaplasia, the Updated Sydney System Biopsy Protocol relies on five gastric biopsies (two from the antrum, one from the incisura angularis, and two from the body) [37]. While this may have contributed to lower overall sensitivity rates, it is unlikely that the differences observed with suspected GI bleeding and prior *H. pylori* infection were affected.

Despite these limitations, our study demonstrates that, compared with PCR, two commonly used invasive diagnostic methods exhibit high specificity but low sensitivity for *H. pylori* detection among patients in this clinical setting. This lower sensitivity is most pronounced among older patients. Specifically, we identified a statistically significant decrease in mean age among patients with true positive test results compared to those with false negative results with both histopathology and a CLOtest. The Maastricht VI/Florence Consensus Report recommends invasive diagnostic testing for *H. pylori* among patients over 50 years of age with symptomatic dyspepsia due to their increased risk of gastric cancer [38]. Accordingly, adherence to this recommendation is likely to yield insufficient *H. pylori* detection among patients in whom these invasive methods are most strongly indicated and may be contributing to the elevated prevalence of *H. pylori* infection and gastric cancer within the Navajo Nation [3,4,5]. One possible contributing factor to this difference may be that older patients exhibit a greater lifetime exposure to heavy metals in a region wherein over 30% of inhabitants lack access to regulated water systems. However, further research is needed to elucidate this possible connection and to identify other factors [16]. Per the Maastricht recommendations, complementary testing with non-invasive methods, including serology or UBT, should be pursued in patients with high clinical suspicion for *H. pylori* infection, even if invasive testing yielded negative findings. Our results also suggest that invasive testing exhibits high specificity for *H. pylori* diagnosis, which, when understood in the context of the Navajo population’s 3–4 times higher disease prevalence compared to non-Hispanic Whites, warrants both diagnostic and therapeutic vigilance on behalf of clinicians to ensure that adequate detection and treatment are undertaken.

## 5. Conclusions

The high specificity of these tests (i.e., false positive results are rare) indicates that emphasis should be placed on ensuring adequate treatment of a suspected positive infection within the Navajo population, given the high *H. pylori* prevalence and limited diagnostic sensitivity. Including a more sensitive test might reduce the number of individuals falsely classified as *H. pylori* negative, especially older patients in whom invasive testing is most indicated. This test limitation should be considered when setting up clinical approaches to serving this population and reducing the burden of gastric disease and gastric cancer.

## Figures and Tables

**Table 1:** Demographic characteristics and symptoms of Navajo patients undergoing their first endoscopy by *H. pylori* status using PCR sequencing.

	Total *	*H. pylori* +	*H. pylori* −	Statistics
	(N = 399)	(N = 108)	(N = 291)	
Age (mean (sd))	56.2 (14.2)	52.5 (15.6)	57.6 (13.5)	t = 3.2, *p* = 0.001
Missing (n = 2)				
Age Group, years (n (%))				X^2^ = 14.6, *p* = 0.006
18–34	39 (9.8%)	18 (16.7%)	21 (7.3%)	
35–44	44 (11.1%)	18 (16.7%)	26 (9.0%)	
45–54	80 (20.2%)	18 (16.7%)	62 (21.5%)	
55–64.	103 (25.9%)	26 (24.1%)	77 (26.6%)	
65+	131 (33.0%)	28 (25.9%)	103 (35.6%)	
Missing (n = 2)				
Tribal Affiliation (n (%))				X^2^ = 3.5, *p* = 0.17
Navajo	377 (95.7%)	102 (96.2%)	275 (95.5%)	
Hopi	14 (3.6%)	2 (1.9%)	12 (4.2%)	
Other	3 (0.8%)	2 (1.9%)	1 (0.4%)	
Missing (n = 5)				
Sex (n (%))				X^2^ = 7.3, *p* = 0.007
Female	290 (72.9%)	68 (63.0%)	222 (76.6%)	
Male	108 (27.1%)	40 (37.0%)	68 (23.4%)	
Missing (n = 1)				
BMI (n (%))				X^2^ = 5.3, *p* = 0.15
Underweight (<18.5)	6 (1.6%)	3 (2.9%)	3 (1.1%)	
Normal (18.5–24.9)	42 (10.8%)	13 (12.4%)	29 (10.3%)	
Overweight (25–29.9)	125 (32.3%)	40 (38.1%)	85 (30.1%)	
Obese (>30)	214 (55.3%)	49 (46.7%)	165 (58.5%)	
Missing (n = 12)				
Current Tobacco Use (smoke &/or smokeless) (n (%))				X^2^ = 0.91, *p* = 0.63
Yes	52 (13.1%)	17 (15.7%)	35 (12.2%)	
No	278 (70.2%)	73 (67.6%)	205 (71.2%)	
Ceremonial Use only	66 (16.7%)	18 (16.7%)	48 (16.7%)	
Missing (n = 3)				
GI Bleed Indicated (anemia, blood in vomit or stool) (n (%))				X^2^ = 0.58, *p* = 0.44
Yes	71 (17.8%)	22 (20.2%)	49 (16.9%)	
No	328 (82.2%)	87 (79.8%)	241 (83.1%)	
Missing (n = 0)				
History of *Helicobacter pylori* infection (n (%))				X^2^ = 1.34, *p* = 0.25
Yes	165 (41.4%)	40 (36.7%)	125 (43.1%)	
No	234 (58.7%)	69 (63.3%)	165 (56.9%)	
Missing (n = 0)				

* Samples with missing test results or age and sex information were excluded during statistical analysis.

**Table 2:** Summary test performance statistics for three *H. pylori* diagnostic tests assuming an underlying prevalence of 23%. PCR of the *H. pylori* 16S gene used as the gold standard.

Diagnostic Test	Sensitivity% (95% CI)	Specificity% (95% CI)	PPV% (95% CI)	NPV % (95% CI)	ROC Area Value (95% CI)	Agreement%	Kappa
Histopathology							
Overall (n = 384)	72.0 (62.5, 80.2)	94.2 (90.8, 96.7)	78.8 (69.5, 85.9)	91.8 (89.2, 93.9)	0.831 (0.786, 0.876)	87.9	0.68, *p* < 0.001
Hp HX * (n = 160)	62.5 (45.8, 77.3)	93.3 (87.3, 97.1)	73.7 (57.9, 85.1)	89.3 (84.8, 92.6)	0.779 (0.700, 0.858)	85.6	0.59, *p* < 0.001
No Hp Hx (n = 224)	77.6 (65.8, 86.9)	94.9 (90.2, 97.8)	82.0 (69.6, 90.0)	93.4 (90.1, 95.7)	0.863 (0.809, 0.916)	89.7	0.77, *p* < 0.001
GI Bleed * (n = 69)	90.5 (69.6, 98.8)	89.6 (77.3, 96.5)	72.2 (52.8, 85.7)	96.9 (89.4, 99.2)	0.900 (0.823, 0.978)	89.9	0.77, *p* < 0.001
No GI Bleed (n = 315)	67.4 (56.5, 77.2)	95.2 (91.6, 97.6)	80.7 (69.8, 88.4)	90.7 (87.8, 93.0)	0.813 (0.761, 0.865)	87.6	0.67, *p* < 0.001
CLO							
Overall (n = 337)	57.0 (45.8, 67.6)	97.2 (94.3, 98.9)	85.9 (74.2, 92.8)	88.3 (85.6, 90.6)	0.771 (0.717, 0.825)	86.9	0.61, *p* < 0.001
Hp HX (n = 146)	50.0 (32.4, 67.6)	97.3 (92.4, 99.4)	84.8 (63.5, 94.7)	86.7 (82.3, 90.1)	0.737 (0.650, 0.823)	86.3	0.55, *p* < 0.001
No Hp Hx (n = 191) *	61.5 (44.0, 74.7)	97.1 (92.8, 99.2)	86.5 (70.4, 94.5)	89.4 (85.7, 92.3)	0.793 (0.725, 0.862)	87.3	0.65, *p* < 0.001
GI Bleed * (n = 63)	66.7 (41.0, 86.7)	91.1 (78.8, 97.5)	69.1 (45.4, 85.8)	90.1 (82.6, 94.7)	0.789 (0.669, 0.909)	84.1	0.60, *p* < 0.001
No GI Bleed * (n = 274)	54.4 (41.9, 66.5)	98.5 (95.8, 99.7)	91.8 (78.0, 97.2)	87.9 (84.9, 90.4)	0.765 (0.705, 0.825)	87.6	0.61, *p* < 0.001
Combination (Histopathology or CLO Positive treated as positive)							
Overall * (n = 397)	72.2 (62.8, 80.4)	94.5 (91.1; 96.8)	79.6 (70.5, 86.4)	91.9 (89.3, 93.9)	0.833 (0.789, 0.878)	88.4	0.70, *p* < 0.001
Hp HX * (n = 165)	65.0 (48.3, 79.4)	93.6 (87.8, 97.4)	75.2 (59.9, 86.0)	90.0 (85.4, 93.2)	0.793 (0.715, 0.871)	86.7	0.62, *p* < 0.001
No Hp Hx * (n = 232)	76.5 (64.6, 85.9)	95.1 (90.6, 97.9)	82.4 (70.2, 90.3)	93.1 (89.8, 95.4)	0.858 (0.805, 0.911)	89.7	0.74, *p* < 0.001
GI Bleed * (n = 70)	95.2 (76.2; 99.9)	89.8 (77.8; 96.6)	73.6 (54.7, 86.5)	98.4 (90.3, 99.8)	0.925 (0.862, 0.989)	91.4	0.81, *p* < 0.001
No GI Bleed * (n = 327)	66.7 (55.7, 76.4)	95.4 (91.9, 97.7)	81.3 (70.5, 88.7)	90.6 (87.7, 92.8)	0.810 (0.759, 0.862)	87.8	0.66, *p* < 0.001

* Calculation included cell counts 8 or less.

## Data Availability

The data presented in this study are available on request from the Navajo Nation Human Research Review Board because they are the property of the Navajo Nation.

## Supplementary Materials

The following supporting information can be downloaded at: https://www.mdpi.com/article/10.3390/microorganisms13081920/s1, Table S1: Demographic and clinical characteristics of false positives and false negatives; Table S2: Comparison of Positive and Negative Predictive Values.
