# Supplementary material for: Helicobacter pylori Diagnostic Testing Accuracy in a High-Prevalence Native American Population of Northern Arizona
Source: Microorganisms. 2025 Aug 18;13(8):1920. doi: 10.3390/microorganisms13081920 (PMC12388262; doi:10.3390/microorganisms13081920)
Supplement: Supplementary file 1 [file microorganisms-13-01920-s001.zip › microorganisms-3722795-supplementary.pdf]

Supplemental materials.

Table S1. Demographic and clinical characteristics of false positives and false negatives.

A) Among PCR Positive

	Histopathology		Statistic	CLO		Statistic
	True Positive (n=76)	False Negative (n=30)		True Positive (n=49)	False Negative (n=36)	
Age			**t = 2.04**			**t = 3.06**
mean(sd)	50.7 (15.2)	57.6 (16.1)	**p=0.04**	49.6 (14.2)	59.6 (15.8)	**p < 0.01**
Missing	0	1		0	0	
Age Group, years (n (%))			X^2 = 8.4			**X2 = 15.2**
18-34	13 (17.1%)	5 (17.1%)	p = 0.07	9 (18.4%)	4 (11.1%)	**p < 0.01**
35-44	14 (18.4%)	3 (10.3%)		8 (16.3%)	4 (11.1%)	
45-54	16 (21.1%)	1 (3.5%)		13 (26.3%)	1 (2.8%)	
55-64	17 (22.4%)	8 (27.6%)		11 (22.5%)	10 (27.8%)	
65+	16 (21.1%)	12 (41.4%)		8 (16.3%)	17 (47.2%)	
Missing	0	1		0	0	
Sex (n (%))			X^2 = 2.50			X2 = 2.0
Female	45 (59.2%)	22 (75.9%)	p = 0.17	28 (57.1%)	26 (72.2%)	p = 0.12
Male	31 (40.8%)	7 (24.1%)		21 (42.9%)	10 (27.8%)	
Missing	0	1		0	0	
BMI (n (%))			X^2 = 4.6			X2 = 1.6
Underweight (<18.5)	2 (2.7%)	1 (3.5%)	p = 0.17	1 (2.1%)	2 (5.7%)	p =0.70
Normal (18.5-24.9)	11 (15.1%)	1 (3.5%)		6 (12.8%)	3 (8.6%)	
Overweight (25-29.9)	24 (32.9%)	15 (51.7%)		15 (31.9%)	14 (40.0%)	
Obese (>30)	36 (49.3%)	12 (41.3%)		25 (53.2%)	16 (45.2%)	
Missing	3	1		2	1	
Current Tobacco Use (n (%))			X^2 = 4.6			**X2 = 6.5**
Yes	15 (19.7%)	2 (6.7%)	p =0.11	11 (22.5%)	1 (2.9%)	**p = 0.03**
No	50 (65.8%)	20 (66.7%)		30 (61.2%)	28 (80.0%)	
Ceremonial Use only	10 (13.2%)	10 (13.3%)		8 (16.3%)	6 (17.1%)	
Missing (n=1)	1	0		0	1	

B) Among PCR Negative

	Histopathology		Statistic	CLO		Statistic
	True Negative (n=261)	False Positive (n=17)		True Negative (n=245)	False Positive (n=7)	
Age			t = -0.20			t = 0.44
mean(sd)	57.3 (13.7)	58 (10.9)	p = 0.84	58.3 (13.3)	56 (14.2)	p = 0.66
Missing (n=1)	1	0		0	0	
Age Group, years (n (%))			X^2 = 3.2			X2 = 1.1
18-34	20 (7.7%)	0	p = 0.64	16 (6.5%)	0	p = 0.82
35-44	25 (9.6%)	1 (5.9%)		20 (8.2%)	1 (14.3%)	
45-54	55 (21.2%)	6 (35.3%)		51 (20.8%)	2 (28.6%)	
55-64	68 (26.2%)	5 (29.4%)		65 (26.5%)	2 (28.6%)	
65+	92 (35.4%)	5 (29.4%)		93 (8.0%)	2 (28.6%)	
Missing (n=1)	1	0		0	0	
Sex (n (%))			X^2 = 0.26			X2 = 1.9
Female	201 (77.0%)	14 (82.4%)	p = 0.77	193 (78.8%)	4 (57.1%)	p = 0.18
Male	60 (23.0%)	3 (17.7%)		52 (21.2%)	3 (42.9%)	
Missing	0	0		0	0	
BMI (n (%))			X2 = 3.5			X2 = 5.0
Underweight (<18.5)	3 (1.2%)	0	p = 0.24	2 (<1%)	0	p = 0.10
Normal (18.5-24.9)	25 (9.8%)	3 (18.8%)		27 (11.3%)	2 (33.3%)	
Overweight (25-29.9)	76 (29.8%)	7 (43.8%)		70 (29.4%)	3 (50%)	
Obese (>30)	151 (59.2%)	6 (37.5%)		139 (58.4%)	1 (16.7%)	
Missing	6	1		7	1	
Current Tobacco Use (n (%))			X^2 = 0.55			X2 = 1.9
Yes	31 (12.0%)		p = 0.67	28 (11.5%)	2 (28.6%)	p = 0.28
No	186 (71.8%)	3 (17.7%)		176 (72.4%)	4 (575.1%)	
Ceremonial Use only	42 (16.2%)	11 (64.7%)		39 (16.1%)	1 (14.3%)	
Missing	2	3 (17.7%)		3	0	

Table S2. Comparison of Positive and Negative Predictive Value, Assuming 65% Prevalence

Because the community prevalence is much higher, we also summarize the PPV & NPV assuming a prevalence of 65%.

	Assuming 23% Prevalence		Assuming 65% Prevalence	
	PPV	NPV	PPV	NPV
Histopathology				
Overall (n=384)	77.8 (68.5, 84.9)	91.7(89.1, 93.8)	95.6 (93.1, 97.2)	64.1 (56.8, 70.8)
Hp HX* (n=160)	73.7 (57.9, 85.1)	89.3 (84.8, 92.6)	94.6 (89.5, 97.3)	57.3 (47.2, 66.7)
No Hp Hx (n=224)	80.2 (67.9, 88.6)	93.3 (89.9, 95.6)	96.6 (93.4, 98.3)	69.5 (59.3, 78.1)
GI Bleed* (n=69)	72.2 (52.8, 85.7)	96.9 (89.4, 99.2)	94.2 (87.4, 97.4)	83.5 (57.5, 95.0)
No GI Bleed (n=315)	79.3 (68.5, 87.2)	90.6 (87.7, 92.9)	96.0 (93.1, 97.7)	60.8 (53.3, 67.8)
CLO				
Overall (n=337)	86.1 (74.5, 92.9)	88.5 (85.7, 90.8)	97.5 (94.8, 98.8)	55.3 (49.1, 61.3)
Hp HX (n=146)	84.8 (63.5, 94.7)	86.7 (82.3, 90.1)	97.2 (91.5, 99.1)	51.2 (42.8, 59.5)
No Hp Hx (n=191)*	86.8 (70.9, 94.6)	89.7 (85.9, 92.6)	97.5 (93.7, 99.1)	57.6 (49.1, 65.8)
GI Bleed* (n=63)	69.1 (45.4, 85.8)	90.1 (82.6, 94.7)	93.3 (83.8, 97.4)	59.5 (43.2, 74.0)
No GI Bleed* (n=274)	91.9 (78.4, 97.3)	88.1 (85.0, 90.6)	98.6 (95.8, 99.6)	54.2(47.6, 60.7)

*Calculation included cell counts 8 or less
